# Global Clinical Practice in Transitioning Protein Substitutes for Children with Phenylketonuria

**DOI:** 10.3390/nu17162650

**Published:** 2025-08-15

**Authors:** Ozlem Yilmaz Nas, Catherine Ashmore, Sharon Evans, Alex Pinto, Anne Daly, Nurcan Yabancı Ayhan, Anita MacDonald

**Affiliations:** 1 Birmingham Women’s and Children’s Hospital, Birmingham B4 6NH, UK; catherine.ashmore@nhs.net (C.A.); sharon.morris6@nhs.net (S.E.); alex.pinto@nhs.net (A.P.); a.daly3@nhs.net (A.D.); anita.macdonald@nhs.net (A.M.); 2 Department of Nutrition and Dietetics, Ankara Yildirim Beyazit University, Ankara 06760, Turkey; 3 Department of Nutrition and Dietetics, Ankara University, Ankara 06290, Turkey; nyabanci@ankara.edu.tr

**Keywords:** transition, protein substitute, international survey, phenylketonuria

## Abstract

**Background:** Protein substitutes are essential in the dietary management of phenylketonuria (PKU). Transition from first-stage phenylalanine (Phe)-free infant formula to second- and third-stage protein substitutes is carefully managed to meet a child’s evolving nutritional needs, feeding abilities, and developmental progression. However, clinical protocols, product access, and reimbursement vary globally. This study assessed international transition practices. **Methods:** A cross-sectional online survey explored health professionals’ practices on transition timing, influencing factors, product forms, casein-glycomacropeptide (cGMP) use, and perceived barriers and facilitators. **Results:** A total of 106 professionals from 32 countries participated: Europe (67%), Asia (12%), North America (10%), South America (8%), and Oceania (3%). Dietitians led transitions in 83% of centers. First-stage Phe-free infant formula was typically discontinued at 1–2 years (66%). Second-stage substitutes were introduced at 6–12 months in Europe (61%) and Oceania (100%), but after age one in Asia (69%), North America (72%), and South America (100%). Influencing factors included weaning alignment (46%) and nutritional needs (42%). Semi-solids were preferred in Europe (56%) and Oceania (67%), while powdered drinks dominated in Asia (62%), North America (82%), and South America (100%). Third-stage protein substitutes were introduced at 3–5 years (45%), with later transitions more common in South America (88%) and North America (63%). Ready-to-drink forms were frequent in Oceania (100%), Asia (92%), and Europe (85%). cGMP was prescribed by 61%, mainly guided by preference, Phe tolerance, and adherence; 26% reported no access. Key facilitators for transition included motivation (79%) and sensory properties (69%); barriers included aversion (70%) and poor taste/texture (69%). School involvement was reported by 32%. **Conclusions:** Protein substitute transition practices in PKU vary globally. International guidance and equitable product access are needed.

## 1. Introduction

Phenylketonuria (PKU, OMIM 261600) is an autosomal recessive disorder caused by a deficiency of the phenylalanine hydroxylase (PAH) enzyme [1,2]. It causes accumulation of phenylalanine (Phe) in the blood and brain, disrupting neurotransmitter production and if untreated leads to cognitive and neuropsychological impairments [3,4,5,6]. Globally, it affects 1 in 23,930 live births, with the highest prevalence (1 in 10,000–15,000) observed in White and East Asian populations [7] and it spans a spectrum of severity. It is historically classified as classical, moderate, mild PKU, or mild hyperphenylalaninemia based on untreated blood Phe levels, but classification may be determined by cofactor responsiveness (e.g., sapropterin) [8,9].

Lifelong management of classical PKU involves a Phe-restricted diet (<10 g/day of natural protein), supplemented with Phe-free amino acid mixtures and/or casein-glycomacropeptide (cGMP) [10], that supply up to 80% of daily protein intake. They commonly contain added carbohydrates, docosahexaenoic acid, vitamins, and minerals [9,11]. The nutritional composition and format of protein substitutes are influenced by the age, developmental stage, and specific needs (e.g., activity levels, pregnancy) of the patient [12,13].

Worldwide, the availability of protein substitutes for PKU varies widely due to differences in the number of patients with PKU, economic conditions, and reimbursement systems [14,15,16,17,18,19]. For example, Pena et al. [16] demonstrated disparities in access to protein substitutes, with the number available ranging from 30 in Turkey to 105 in Germany, reflecting variations in government policies and reimbursement strategies. In most European countries, costs are covered by government funding or health insurance [14,20]. However, in Brazil and China, economic constraints and the absence of national reimbursement systems, lead to limited access to protein substitutes [21,22,23]. In regions with inadequate or non-existent reimbursement systems, companies may be reluctant to introduce a broad range of products, further restricting patient access [24]. Additionally, in many countries, the absence of appropriate weaning protein substitutes [16] commonly leads to healthcare professionals relying on concentrating infant formulas, thereby increasing the content of Phe-free amino acids for older children with PKU. These products are not designed for older age groups and may fail to meet their evolving nutritional needs.

We refer to “transition” as the structured progression from phenylalanine-free infant formula to protein substitutes tailored for older children. This shift reflects evolving nutritional requirements and developmental milestones, ensuring continuity of metabolic control while supporting growth, encouraging feeding autonomy, and promoting age-appropriate dietary practices. In the UK, a structured transition process for protein substitutes is established. This involves a gradual progression from first-stage Phe-free infant formula to a second-stage weaning protein substitute from 6 months of age [25], followed by a transition to third-stage liquid or powdered protein substitutes at around 3 to 5 years of age [26,27]. This system has evolved through clinical practice and research, emphasizing the importance of gradual transitions to meet the changing nutritional and developmental needs of children with PKU. Internationally, there is considerable variability in approaches to transitioning protein substitutes, which can have lasting impacts on patient outcomes. Inconsistent protocols may lead to nutritional inadequacies during critical developmental periods, disrupt the establishment of long-term dietary habits, and negatively influence patient attitudes and adherence to protein substitute regimens throughout life. Understanding variations in practice and their implications is important for optimizing PKU management worldwide. This study aims to assess global practices and its application for transitioning protein substitutes and any challenges this presents in children with PKU.

## 2. Methodology

### 2.1. Study Design and Questionnaire

This global cross-sectional study collected data from healthcare professionals using a non-validated, 30-item online questionnaire developed on Google Forms by the research team (O.Y.N. and A.M.). The questionnaire, administered between March and April 2023, included 17 multiple-choice and 13 open-ended questions. To ensure clarity and consistency in responses, we provided definitions and examples of different types of protein substitutes. Specifically, the transition stages were outlined, i.e., first-stage Phe-free infant formula, second-stage weaning protein substitutes (e.g., spoonable gels, low-volume liquids), and third-stage protein substitutes (e.g., ready-to-drink liquids, powders, or tablets). Respondents were therefore given a standardized explanation of a transition process in order to enhance the reliability and comparability of responses and minimize any misunderstanding.

The questionnaire (Supplementary File S1) explored several key areas: (1) clinic characteristics, including location, patient numbers, and the types of patients (pediatric/adult); (2) transition practices for protein substitutes, such as the timing of transition, factors influencing timing, product types, aspects affecting the selection of protein substitutes, the transition approach (e.g., stepwise, immediate, individualized), duration and perceived difficulty of the transition, subsequent changes in third-stage protein substitutes (including use of cGMP prescriptions), follow-up communication, and the involvement of nursery or school staff; (3) any factors that support or challenge the transition process, along with potential solutions or improvement strategies for enhancing the process; and (4) differences in transition practices across non-PKU protein metabolism disorders.

### 2.2. Recruitment of Healthcare Professionals

A total of 298 dietitians and doctors involved in PKU care worldwide were invited to participate in the questionnaire. Invitations were distributed through professional networks and other relevant clinical and research collaborations. To ensure diverse representation, multiple professionals from the same center were encouraged to participate. Recruitment was further promoted through LinkedIn and X (formerly Twitter) posts and shared in 12 languages (English, Spanish, Portuguese, Turkish, German, Dutch, Persian, Arabic, Czech, Hungarian, Italian, and French), although the questionnaire itself was available only in English. Three reminders were sent (two weeks prior, one week prior, and on the final day before the survey closed).

### 2.3. Data Analysis

Of the 110 responses received, 106 were retained for analysis after excluding duplicates (e.g., near-identical responses from the same center). Responses to single, top-three-choice, and multiple response questions were summarized as percentages of respondents and were analyzed quantitatively using descriptive statistics in DataTAB version 2025 [28]. For open-ended questions, responses were manually reviewed and divided into common themes. Two of the investigators (O.Y.N. and A.M.) selected illustrative quotes from the responses to open-ended questions. Partial non-responses were addressed by reporting variable denominators (e.g., *n* = 94–106) for individual questions.

### 2.4. Ethics

Ethical approval was not required for this study, as it involved the collection of anonymized, non-sensitive data from healthcare professionals. Informed consent was implied by voluntary participation: respondents were informed of the study’s purpose at the start of the questionnaire, and submission of responses was considered consent. All identifying details (e.g., names of individuals, hospitals, or institutions) were removed to ensure confidentiality. Data were stored securely and accessed only by the research team.

## 3. Results

### 3.1. Participants

A total of 106 healthcare professionals from 32 countries across five continents participated in the study:

Europe (*n* = 71, 67%): Belgium (*n* = 4), Cyprus (*n* = 2), Denmark (*n* = 3), France (*n* = 4), Germany (*n* = 7), Greece (*n* = 1), Italy (*n* = 7), Latvia (*n* = 1), Moldova (*n* = 1), Netherlands (*n* = 3), Norway (*n* = 1), Portugal (*n* = 3), Romania (*n* = 1), Slovenia (*n* = 2), Spain (*n* = 9), Sweden (*n* = 2), Switzerland (*n* = 3), UK (*n* = 17).

Asia (*n* = 13, 12%): Iran (*n* = 1), Qatar (*n* = 1), Saudi Arabia (*n* = 2), Singapore (*n* = 1), Turkey (*n* = 7), United Arab Emirates (*n* = 1).

North America (*n* = 11, 10%): Canada (*n* = 1), USA (*n* = 10).

South America (*n* = 8, 8%): Argentina (*n* = 3), Brazil (*n* = 4), Chile (*n* = 1).

Oceania (*n* = 3, 3%): Australia (*n* = 2), New Zealand (*n* = 1).

The majority of respondents (69%, *n* = 73/106) reported that their centers treated both adults and children with PKU, while the remaining centers (31%, *n* = 33/106) treated only children. Across all centers, the median number of patients with PKU was 100 (Q1: 43, Q3: 170), with a median of 54 patients per center (Q1: 28, Q3: 100) under 16 years of age. Eighty-three percent of healthcare professionals (*n* = 88/106) identified dietitians as primarily leading the transition process, 9% (*n* = 10/106) were medical doctors, and 8% (*n* = 8/106) reported a joint approach.

### 3.2. Global Transition Practices from First-Stage Phe-Free Infant Formula to Second-Stage Protein Substitutes

#### Timing, Influencing Factors, and Product Forms

The transition from first-stage Phe-free infant formula to second-stage weaning protein substitutes varied globally in timing, influencing factors, and product format or type (Table 1).

Most respondents (66%, *n* = 68/103) discontinued first-stage Phe-free infant formula between 1 and 2 years of age, although some discontinued earlier (3–5 months: 1%, *n* = 1/103, 6–12 months: 22%, *n* = 23/103) or later (after 2 years: 11%, *n* = 11/103).

The introduction of second-stage weaning protein substitutes was most common at 6–12 months (50%, *n* = 53/105) but also occurred earlier (3–5 months: 10%, *n* = 10/105) or later (1–2 years: 25%, *n* = 26/105; after 2 years: 15%, *n* = 16/105). Of 99 respondents, the most common reason was alignment with weaning practices (46%, *n* = 46/99), coinciding protein substitute introduction with solid food transition. This was followed by consideration of nutritional requirements (42%, *n* = 42/99), reflecting the need for higher amounts of daily protein equivalent but in a small volume. The most commonly used form of second-stage protein substitutes was powder reconstituted into a drink (52%, *n* = 55/106), followed by semi-solid (43%, *n* = 46/106).

Regional protein substitute prescription patterns:

**Europe:** Most respondents (61%, *n* = 43/70) introduced second-stage weaning protein substitutes at 6–12 months of age, with fewer transitions at 3–5 months (14%, *n* = 10/70), 1–2 years (14%, *n* = 10/70), or after 2 years (10%, *n* = 7/70). The main reasons for transitioning were alignment with weaning practices (60%, *n* = 39/65) and nutritional requirements (40%, *n* = 26/65). Many professionals noted that this transition coincided with solid food introduction, as one respondent explained: *“It is the age when vegetables, fruits, and low-protein foods are introduced.”* Another professional noted: *“The first-stage Phe-free infant formula no longer provides sufficient protein equivalent at that age.”*

The type of second-stage protein substitute most commonly used was powder prepared as a semi-solid (56%, *n* = 40/71)**,** and 41% (*n* = 29/71) used powder as a drink.

**Asia:** The timing of the introduction of second-stage protein substitutes varied, with 31% (*n* = 4/13) between 6 and 12 months, 31% (*n* = 4/13) between 1 and 2 years (*n* = 4/13), and 38% (*n* = 5/13) after 2 years of age. The primary factors influencing this timing were alignment with nutritional requirements (33%, *n* = 4/12) and better acceptance with age (17%, *n* = 2/12). One professional explained: *“Once complementary foods are introduced, many infants struggle to consume enough first-stage formula to meet their daily Phe-free protein needs. Therefore, protein substitutes that provide more protein in less volume are preferred.”* Reimbursement policies (17%, *n* = 2/12) also influenced decision-making, as noted by one professional: *“It is dependent on health insurance regulations.”*

Powders reconstituted into drinks were the most commonly used (62%, *n* = 8/13), while 23% (*n* = 3/13) of respondents used powder reconstituted into a semi-solid format.

**North America:** Second-stage protein substitutes were introduced at 6–12 months (27%, *n* = 3/11), 1–2 years (36%, *n* = 4/11), or after 2 years (36%, *n* = 4/11). The main influencing factor was changing nutritional requirements (64%, *n* = 7/11), as one respondent stated: *“Their protein needs increase, but they do not require as many calories.”* Weaning practices also played a role (36%, *n* = 4/11), as another professional highlighted: *“It aligns with the period when children typically transition from formula to cow’s milk and increase their solid food intake.”* Additionally, 3 (27%) professionals noted that a child’s decreasing interest in Phe-free infant formula contributed to the transition: *“…decreasing interest in Phe-free infant formula as the child grows.”*

The form of second-stage substitutes used in North America was mostly (82%, *n* = 9/11) powder reconstituted as a drink.

**South America:** Second-stage protein substitutes were introduced at 1–2 years in all cases (100%, *n* = 8/8), with no reports of earlier or later introduction. The decision was primarily based on clinical protocols and manufacturer instructions (50%, *n* = 4/8) or nutritional requirements (38%, *n* = 3/8). One respondent explained: *“Due to the manufacturer’s recommendations and the nutritional composition, it meets the needs of children in this age group.”* Another professional emphasized the combined influence of clinical monitoring and product composition, stating: *“We monitor the child’s weight and growth before advancing formulas, but two years is the limit due to differences in micronutrient and amino acid composition in the formulas available in Brazil.”* Volume considerations also influenced the transition: *“We transition at 12 months because most first-stage formulas are designed for use up to that age, and the required volume becomes impractical.”*

Powder prepared as a drink was the only form reported by all professionals (100%, *n* = 8/8).

**Oceania:** Second-stage protein substitutes were introduced between 6 and 12 months of age by all respondents (100%, *n* = 3/3), primarily to align with weaning practices (67%, *n* = 2/3) and meet nutritional requirements (67%, *n* = 2/3). One emphasized: *“Reducing bottle feeds as solids are introduced.”* Another professional highlighted the importance of meeting Phe-free protein requirements while reducing volume: *“Ensuring adequate Phe-free protein intake in a lower-volume feed.”*

Powder reconstituted as a semi-solid was the most common formulation (67%, *n* = 2/3), while one professional (33%, *n* = 1/3) reported using powder as a drink.

### 3.3. Global Transition Practices from Second-Stage to Third-Stage Protein Substitutes

#### 3.3.1. Timing, Influencing Factors, and Product Forms

The transition from second-stage weaning to third-stage protein substitutes varied globally in terms of timing, influencing factors, and product forms (Table 2).

Most respondents (45%, *n* = 46/102) introduced third-stage protein substitutes between 3 and 5 years of age, with others introducing at 6–10 years (31%, *n* = 32/102), after 10 years (14%, *n* = 14/102), or 1–2 years (7%, *n* = 7/102). The timing of this transition was primarily influenced by nutritional and clinical considerations (42%, *n* = 43/102), practical and child behavioral factors (40%, *n* = 41/102), as well as clinical protocols and manufacturer instructions (20%, *n* = 20/102). The most commonly used forms of third-stage protein substitutes worldwide were ready-to-drink liquids (78%, *n* = 82/105), followed by powders prepared as a drink (54%, *n* = 57/105).

**Europe:** The majority of respondents (53%, *n* = 36/68) introduced third-stage protein substitutes at 3–5 years, followed by 6–10 years (30%, *n* = 20/68). One respondent emphasized the importance of convenience: *“It is more practical around the time of nursery and school and provides greater convenience for families.”* Another highlighted growing independence: *“The children are becoming more independent and want more flexibility in their therapy.”* Taste preferences also played a role, as one professional noted: *“By this age, patients often prefer ready-to-drink options or have flavour fatigue and are ready to try something different.”* Additionally, nutritional composition was important: *“The pre-measured sachets contain the right amount of protein equivalent in each dose for older children, and the microelements/vitamins are appropriate for this age period.”*

**Asia:** The age of introduction of third-stage protein substitutes varied, with 3–5 years being the most commonly reported (50%, *n* = 6/12), followed by 6–10 years (17%, *n* = 2/12) and after 10 years (17%, *n* = 2/12). Factors influencing timing of the transition were equally mixed between nutritional considerations, child behavior, protocols and manufacturer instructions, and availability of products. One professional explained: *“Children consume ready-to-drink formulas more easily in places such as kindergartens.”* Another highlighted clinical and manufacturer guidance: *“We give according to manufacturer guidelines, clinical condition of patients, and doctor’s orders.”* Taste was also a decisive factor: *“Patients are not able to accept the taste and smell of second-stage protein substitutes and want flavoured formulas.”* However, limited experience with available formulations posed challenges: *“In our country, powder made into a semi-solid is new, so we have inadequate experience.”*

Ready-to-drink liquids were the most frequently reported form (92%, *n* = 11/12), followed by powders prepared as drinks (50%, *n* = 6/12). No respondents reported using tablets or granules/microgranules.

**North America:** The introduction of third-stage protein substitutes most commonly occurred at 6–10 years (36%, *n* = 4/11) and after 10 years (27%, *n* = 3/11). Nutritional and clinical considerations (91%, *n* = 10/11) were primary drivers. One respondent noted: *“More protein in a smaller volume is needed as children grow.”* Another emphasized that third-stage substitutes are designed for older children: *“Most third-stage protein substitutes are marketed for children over 9 years of age, with nutrient profiles tailored to meet their needs.”* Convenience at school was a key factor: *“Kids often do better with lower volume, GMP, or ready-to-drink options at school.”* Taste preferences and hunger management also played a role: *“Taste preference or patient complaining of hunger often drives the transition.”*

Powders prepared as drinks were the most commonly used form (91%, *n* = 10/11), followed by ready-to-drink liquids (73%, *n* = 8/11). Tablets, bars, and granules/microgranules were not reported.

**South America:** Most transitions occurred at 6–10 years (63%, *n* = 5/8), followed by after 10 years (25%, *n* = 2/8). Nutritional and clinical considerations (50%, *n* = 4/8), protocols and manufacturer instructions (38%, *n* = 3/8), and practical reasons (25%, *n* = 2/8) influenced timing. One professional explained: *“We observe the child’s weight to carry out the evolution of the formula, but 10 years is the limit due to the difference in micronutrients and amino acids between the formulas sold in Brazil.”* Manufacturer recommendations also guided transitions: *“Due to the manufacturer’s indication and the nutritional composition of the formula, it meets the demands of children in this age group.”* Taste and practicality were additional considerations: *“We make the change when the second-stage formulas we use require it, focusing on improving taste and reducing the amount of powder.”*

All respondents (100%, *n* = 8/8) used powders as drinks, with one professional (13%) also using tablets. No respondents used ready-to-drink liquids, bars, semi-solid powders, or granules.

**Oceania:** The introduction of third-stage protein substitutes varied among respondents, with one reporting 1–2 years, another reporting 3–5 years, and the third reporting 6–10 years. One respondent emphasized school readiness: *“Preparation for school is a key factor in the transition.”* Another highlighted reimbursement policies: *“The PBS (Pharmaceutical Benefits Scheme) listing is for children over 12–24 months for most third-stage products.”* One professional found the transition practical despite limited product options: *“The limited product range and flavours available in our country still work for our patients.”*

There was no use of tablets, bars, semi-solid powders, or granules/microgranules.

#### 3.3.2. Duration and Perceived Difficulty of the Transition Process

The duration of transition to third-stage protein substitutes varied by continent, with most transitions occurring within 2 months globally (59%, *n* = 55/94) (Table 3). Healthcare professionals rated the transition process as follows: 43% (*n* = 46/106) described it as normal, 35% (*n* = 37/106) as easy or very easy, 19% (*n* = 20/106) as difficult, and 3% (*n* = 3/106) as very difficult.

#### 3.3.3. Transition Methods

Globally, the most common method for transitioning to third-stage protein substitutes was the step-by-step approach (41%, *n* = 41/99), followed by individualized approach (31%, *n* = 31/99) and immediate transitions (27%, *n* = 27/99).

**Europe:** The step-by-step method was most prevalent (43%, *n* = 29/67), followed by individualized approach (30%, *n* = 20/67) and immediate changeovers (27%, *n* = 18/67). Many respondents favored a gradual introduction, with one professional explaining: *“The approach is step by step. The liquid protein substitute is introduced once daily in addition to the second-stage protein substitute. It is gradually increased week by week until a full pouch is consumed, at which point it replaces one dose of the second-stage substitute. The second and third doses are not changed over until the child is fully and comfortably established on one dose.”* Another respondent described a similar process: *“We add an additional 10 mL of liquid protein substitute, gradually increasing until we achieve one full dose, and then change one dose at a time.”* Individualized transitions were also common, as noted by one respondent: *“Dependent on the patient and family. Some are happy to immediately switch, but most require a more step-by-step approach, which takes time.”*

**Asia:** Both step-by-step (42%, *n* = 5/12) and immediate transitions (42%, *n* = 5/12) were common, with an individualized approach being less frequent (16%, *n* = 2/12). One respondent noted: *“We replace one feed at a time, starting with the minimum amount and gradually increasing after the patient tolerates this quantity.”* Immediate transitions occurred when families were prepared, with one respondent adding: *“Immediate changeover. After the family procures the new protein substitute, the dietary program is prepared accordingly, and the transition is provided within 1–2 days.”*

**North America:** Individualized transitions were the most common (64%, *n* = 7/11), followed by step-by-step (27%, *n* = 3/11) and immediate changeovers (9%, *n* = 1/11). Patient preferences were important in determining the method. One respondent shared: *“Patient-led—if trying a completely new formula, most patients prefer an immediate changeover at this age.”* The step-by-step method was also used for transitioning to new formulas. One respondent explained the approach: *“Step 1: Start with 25% new formula and 75% old formula. Step 2: Increase to 50/50 new and old. Step 3: Mix 25% old formula with 75% new formula. Step 4: 100% new formula.”* However, some families preferred to keep both second- and third-stage products together to offer variety and reduce volume. As one respondent noted: *“Sometimes, the family wants to keep the stage two and three products together for variety and a decrease in volume.”*

**South America:** Many professionals (67%, *n* = 4/6) emphasized step-by-step transitions, while the remaining 33% (*n* = 2/6) preferred immediate transitions. One professional shared: *“I discuss the transition with the mother or guardian during the consultation, and we start the process with 25%, 50%, and 75% of the prescribed protein requirement until we completely replace the second-stage product.”* One respondent explained that immediate transition was common due to the similarity between products: *“Usually a direct change is made since in our country only powdered protein substitutes are available, and the nutritional composition (as well as the taste) of second- and third-stage products are very similar.”*

**Oceania:** Individualized approach was common (67%, *n* = 2/3), with one respondent (33%, *n* = 1/3) using immediate transition, depending on the patient’s needs. One respondent noted: *“We do both and discuss options with the family—immediate or step-by-step.”* Another emphasized that the decision was often guided by the child’s and family’s preferences: *“It depends on the child or family.”*

#### 3.3.4. Factors Influencing Choice of Third-Stage Protein Substitutes

Professionals reported the top-three factors influencing their choice of third-stage protein substitutes, with regional differences noted (Table 4). Patient preference was the most common factor (65%, *n* = 69/106), being particularly high in Europe (66%, *n* = 47/71), North America (91%, *n* = 10/11), and Oceania (100%, *n* = 3/3). Convenience of minimal preparation also emerged as a key factor (65%, *n* = 69/106), most notably in Europe (70%, *n* = 50/71), Asia (62%, *n* = 8/13), and Oceania (100%, *n* = 3/3). Nutritional composition of products was the third most influential factor (52%, *n* = 55/106), with the highest percentages in North America (82%, *n* = 9/11) and Asia (54%, *n* = 7/13).

#### 3.3.5. Subsequent Changes in Third-Stage Protein Substitutes

More than half of the healthcare professionals (52%, *n* = 55/106) reported changing to a different third-stage protein substitute after the initial transition. The primary reasons for this change were child resistance due to taste, smell, or texture (80%, *n* = 44/55), excessive volume requirements (56%, *n* = 31/55), supply issues (49%, *n* = 27/55), and gastrointestinal problems (44%, *n* = 24/55). Details are provided in Table 5.

#### 3.3.6. Follow-Up Communication During the Transition Process

Follow-up during the transition to third-stage protein substitutes was predominantly carried out through phone or video calls (76%, *n* = 81/106) and clinic visits (65%, *n* = 69/106). Email communication was used by 30% (*n* = 32/106), while home visits (5%, *n* = 5/106) and school visits (3%, *n* = 3/106) were less frequently reported. Electronic medical record messaging was used by 3% (*n* = 3/106).

#### 3.3.7. The Role of Nursery/School During the Transition Process

About one-third of healthcare professionals (32%, *n* = 34/106) reported receiving support from nursery or school staff during the transition to third-stage protein substitutes, while the majority (68%, *n* = 72/106) did not use this type of support.

Perspectives on school involvement varied. Parents were generally responsible for communication with schools or nurseries, with one professional stating: *“It is always the parent who is responsible for it.” Instructions were typically relayed by families, and healthcare professionals reported only stepping in upon request: “We only contact them when the family asks us to.”* In some cases, nurseries and schools were not involved at all: *“We do not involve nurseries.”* Even when involvement did occur, it was often delayed until after the child had fully adjusted to the new protein substitute: *“Involvement usually happens after the child is fully adjusted.”*

In contrast, other respondents saw value in involving nurseries or schools in protein substitute transition, particularly in cases where children respond better in those settings. For instance: *“Children sometimes accept products better at nursery.”* Others highlighted the role schools can play in supporting the process, provided they receive adequate information: *“We go to schools and talk to teachers to ask them for help in following the guidelines.”* In some settings, healthcare teams maintained close contact with families to support school engagement. One respondent shared: *“Our metabolic team’s dietitian is in close contact with the parents. They manage school staff and caregivers.”* Where engagement occurred, some teams offered formal communication tools: *“We provide a school info pack, liaise and visit the school as needed.”* Others noted that some schools or daycare establishments were willing to accommodate protein substitute consumption: *“If the patient prefers to drink formula in the nursery, schools will participate.”*

#### 3.3.8. Prescription of cGMP Protein Substitutes During Transition

Table 6 presents the cGMP-based protein substitute prescription practices of healthcare professionals. Of the 106 respondents, 61% (*n* = 65/106) prescribed cGMP-based protein substitutes, 26% (*n* = 27/106) reported unavailability of cGMP in their country, and 13% (*n* = 14/106) preferred not to prescribe them. Among the 74 professionals with access to cGMP substitutes (excluding 5 who did not provide details), the key factors influencing prescription were patient preference (43%, *n* = 32/74), Phe tolerance (32%, *n* = 24/74), and adherence issues with amino acid-based substitutes (31%, *n* = 23/74).

Regional variations were observed as follows:

**Europe:** In Europe, 72% (*n* = 51/71) of healthcare professionals prescribed cGMP protein substitutes, 11% (*n* = 8/71) reported unavailability, and 17% (*n* = 12/71) chose not to prescribe them. One respondent shared that cGMP substitutes are often selected when patients struggle with the taste of traditional amino acid-based substitutes: *“We choose GMP as a therapeutic alternative when the amino acid protein substitute is not well accepted by the patient.”* However, Phe tolerance was a critical factor, especially in younger children or those with stricter dietary restrictions, as one professional explained, *“cGMP products contain Phe and may affect blood Phe control in young children.”* One respondent noted: *“We start using GMP at the earliest in adolescents, because of the small amount of Phe the product still contains.”*

**Asia:** All respondents (100%, *n* = 13/13) reported the unavailability of cGMP-based protein substitutes.

**North America:** cGMP-based protein substitutes were prescribed by 82% (*n* = 9/11), while 18% (*n* = 2/11) chose not to prescribe them. cGMP was selected when patients struggle with the traditional amino acid-based protein substitutes. As one respondent mentioned: “*If compliance with amino acid-based products is an issue, we often trial cGMP.*”, while another noted: *“Flavour acceptance typically overrides the cGMP discussion.”* In contrast, another professional explained, *“The impact cGMP can have on a patient’s diet is considerable, as it may contribute up to half of their protein tolerance, affecting diet variety and quality.”* Satiety and gastrointestinal tolerance also play a role, with some respondents highlighting cGMP’s potential to improve satiety and palatability.

**South America:** In South America, cGMP protein substitutes were not widely available, with only Argentina (*n* = 3/8, 38%) reporting their use. Respondents from Brazil and Chile reported the unavailability of cGMP in their countries. Argentine professionals reported taste, ease of use, and microbiota benefits as key reasons for prescribing cGMP. One respondent stated: *“cGMP has a very good taste, is easy to drink, and enhances microbiota.”*, while another emphasized its role in improving adherence: *“cGMP improves adherence due to its smell, palatability, and easy-to-use presentations.”* It was stated cGMP was used alongside amino acid-based protein substitutes, and not as a complete replacement: *“We usually don’t replace all amino acid substitutes with cGMP, just a part.”*

**Oceania:** Of the 3 professionals, 2 (67%) from Australia prescribed cGMP-based protein substitutes, while 1 (33%) from New Zealand reported cGMP unavailability. One respondent explained that the decision to prescribe cGMP is influenced by several factors: *“It depends on protein tolerance, any formula refusal, control, and patient interest.”* Another emphasized the importance of BH4 responsiveness, stating: *“If BH4 responsive, we offer GMP as the formula. If not responsive, we only offer it if there is poor acceptance of amino acid formulas and discuss reducing natural protein intake if Phe levels increase after cGMP introduction.”*

#### 3.3.9. Facilitators and Barriers

Healthcare professionals identified several facilitators and barriers in the transition from second- to third-stage protein substitutes (Table 7). The most frequently reported facilitators included child and parental motivation (79%, *n* = 84/106), improved sensory properties of the third-stage protein substitutes (69%, *n* = 73/106), and effective parental management strategies (57%, *n* = 60/106). Conversely, several challenges were reported, with the most common being child resistance to the new protein substitute (70%, *n* = 74/106) and dissatisfaction with its taste, smell, or texture (69%, *n* = 73/106). Additional barriers included parental inconsistency (32%, *n* = 34/106), fear of change (31%, *n* = 33/106), and behavioral difficulties in children (25%, *n* = 26/106).

### 3.4. Suggestions for Supporting the Transition Process

Healthcare professionals suggested various tools and strategies to improve the transition from second-stage to third-stage protein substitutes. The most frequently recommended support tool was educational videos (51%, *n* = 54/106), followed by step-by-step charts or books (39%, *n* = 41/106) and guidance books (39%, *n* = 41/106). Child rewards or sticker charts were suggested by 20% (*n* = 21/106), while extra support, such as peer support, home visits, or follow-ups were highlighted by 18% (*n* = 19/106).

Visual and digital resources were regarded highly for their efficiency and accessibility, with one professional noting: *“Resources need to be simple and quick to read, as lengthy documents do not get read.”* Some suggested digital alternatives to enhance engagement, such as *“an app for children with rewards as they progress through the transition process.”* Some professionals emphasized that *“there is no one-size-fits-all solution”* due to the varying needs of children and families.

In countries with limited protein substitute options, concerns were raised, with one professional stating, *“We don’t have options for protein substitutes in Brazil.”* Some respondents pointed to systemic and logistical barriers, including reimbursement policies and communication delays. Additionally, there were calls for clearer, parent-friendly communication to *“demystify differences between products and explain the need to change over time.”*

### 3.5. Protein Substitute Transition Process Across Different Disorders of Protein Metabolism

We asked healthcare professionals if the transition process for protein substitutes is similar for other disorders of protein metabolism. The majority (65%, *n* = 68/105) reported that it is the same, while 35% (*n* = 37/105) noted differences. Professionals highlighted disparities in protein substitute options, with one respondent stating: *“We don’t have enough options for other protein metabolism disorders, while PKU offers a wider range.”* Another shared: *“PKU patients often experience smoother transitions due to more product options and patient events, while non-PKU patients face challenges such as limited product availability.”* Some noted that non-PKU patients may be tube-fed, as one respondent explained: *“In other conditions, children might be tube-fed, and there’s less variety of products.”* Differences in clinical needs were also emphasized: *“In MSUD, transitions can take longer due to fear of losing metabolic control and food phobia.”*

## 4. Discussion

This survey is the first to report global clinical protein substitute transition practices in PKU. The findings describe wide international variation in transition timing, protein substitute types, and clinical approaches, shaped by regional protocols, reimbursement policies, and product availability. Dietitians led the transition in 83% of centers. Most professionals reported discontinuing the first-stage Phe-free infant formula between 1 and 2 years of age. Second-stage protein substitutes were commonly introduced during infancy in Europe and Oceania, but more often after the first year of life in Asia, North America, and South America. Third-stage protein substitutes were most frequently introduced at 3–5 years, with later use beyond 6 years more common in South America and North America. Some regions reported access to a broad range of protein substitutes including semi-solids, ready-to-drink liquids, and cGMP, while others faced more limited choices. Only a minority of professionals reported school or nursery involvement during protein substitute transition, with most communication led by parents. Healthcare professionals identified both barriers and facilitators to successful transition processes and emphasized the need for practical tools to support families. The findings highlight the lack of global standardization in protein substitute transition and the need for evidence-based guidance and equitable product access.

Regional practices varied widely in both the timing and forms of protein substitutes used during the transition from first-stage Phe-free infant formula to second-stage weaning protein substitutes. In Europe and Oceania, second-stage protein substitutes were typically introduced during infancy, with semi-solid forms commonly used. This approach aligns with PKU weaning recommendations, which encourage early introduction of more concentrated, lower-volume protein substitutes to reduce reliance on liquid infant formula and support appetite and feeding progression [25]. In contrast, in South America, introduction often occurred later, between 1 and 2 years of age, with reliance on powdered drinks, possibly due to the unavailability of semi-solid options. This may contribute to feeding difficulties, as large volumes of Phe-free liquid supplements have been associated with adverse responses such as crying, gagging, screaming, and protein substitute refusal [29]. A longitudinal study in PKU [30] further highlights that the period between 12 and 18 months of age is particularly challenging for families, with increased maternal stress, difficulties in protein substitute administration, and increased blood Phe levels during teething and illness. Health professionals should be aware of this period and offer proactive, structured support to help reduce caregiver burden. These findings highlight the need for international guidance that promotes timely transition to second-stage protein substitutes and ensures equitable access to varied product forms. Further studies are needed to assess how the timing and form of second-stage protein substitutes affect long-term acceptance, adherence, and parental wellbeing.

Third-stage protein substitutes are available in a variety of forms, including powders, ready-to-drink liquids, cGMP-based products, tablets, and slow-release granules, with recommended introduction ages ranging from preschool to adolescence [26,31,32]. In this study, reported transition ages to third-stage protein substitutes ranged from 1 to 2 years to over 10 years. In Europe and Asia, most professionals introduced third-stage protein substitutes between ages 3 and 5, often around the time of growing feeding independence and school entry. This aligns with previous research reporting a median transition age of 4.8 years linked to nursery or school enrolment [26]. In contrast, later introduction was more common in North and South America, with timing influenced by clinical, practical, and product-availability factors. One respondent from North America noted that many third-stage formulations are marketed from age nine, which may contribute to delayed use and reflects regional differences in manufacturer guidance and product formulation. These findings suggest that commercial factors, such as product availability, age suitability labeling, and promotion, may also influence transition practices, highlighting the need to align marketing strategies with children’s developmental and nutritional requirements.

Preferred third-stage protein substitute types varied by region. Ready-to-drink liquids were the most commonly used globally, particularly in Europe, Asia, and Oceania. These products are generally favored for their convenience, portability, and increased flavor options [33]. Powders prepared as drinks were also widely used, especially in North and South America, where they were often the primary option. Other forms such as semi-solids, granules, tablets, or bars were rarely used. South America was the only region without access to ready-to-drink forms, with limited product availability reported as the main factor influencing choices. A previous study [16] similarly showed wide variation in the number of available protein substitutes across Europe. In this study, some health professionals also noted that individuals with other protein metabolism disorders had even fewer protein substitute options than those with PKU, as choices were often restricted by clinical needs or product availability. These findings highlight the need to expand access to a broader range of third-stage protein substitutes and to address persistent barriers related to reimbursement and distribution across all regions and protein metabolism disorders.

Transition methods from second- to third-stage protein substitutes varied across regions, with step-by-step transition being the most common globally, followed by individualized and immediate approaches. Although most professionals completed the transition within six months, both the methods and timing showed regional variation. In Europe, gradual and individualized transitions were commonly used, often involving a progressive adjustment of the third-stage protein substitute dose until full replacement was achieved. This aligns with a previous single-center UK study, suggesting that stepwise transition can support a successful transition in young children with PKU, especially when caregiver consistency and school support are present [27]. In Asia, both step-by-step and immediate transitions were commonly reported, with the latter often occurring when families felt prepared for the change. In South America, gradual transitions were generally preferred, although immediate transitions were sometimes used due to the similarity in nutritional content and format between second- and third-stage powdered substitutes. In North America, individualized transitions predominated, shaped by patient preferences and a flexible approach, with some families choosing to alternate or combine products to increase variety or reduce volume. In Oceania, transition methods were typically tailored to each child’s and family’s needs, with both immediate and gradual approaches used depending on the situation. While nutritional and clinical considerations were the most frequently reported factors influencing timing, decisions were also shaped by product availability, clinician judgment, and the preferences and readiness of families and children.

Use of cGMP-based protein substitutes varied by region. These products were most commonly prescribed in North America and Europe, often used when adherence to amino acid-based protein substitutes was poor. Some healthcare professionals used cGMP as a partial replacement, adjusting natural protein intake accordingly. However, concerns about residual Phe content led to more cautious use in younger children or those with low Phe tolerance. In contrast, cGMP was not available in Asia and in parts of South America and Oceania. Improving global access to cGMP-based protein substitutes, along with clear clinical guidance, is particularly important in cases of poor adherence, especially among older children and adolescents.

Schools and nurseries can play an important role in supporting the transition to third-stage protein substitutes. A previous study showed that children attending full-time nursery experienced smoother transitions, likely due to the structure, routine, and support provided in those settings [27]. However, in this study, school involvement was generally limited. Communication was typically parent-led, with healthcare professionals involved only upon request. Several respondents noted that contact with schools often occurred after the child had already adjusted to the new protein substitute, suggesting a reactive rather than proactive approach. Strengthening collaboration between healthcare teams and educational settings may help improve transition outcomes. Effective models include early communication, staff training, and individualized transition plans [27,34,35].

Key facilitators of a successful transition included child and caregiver motivation, improved taste and texture of the next protein substitute, and consistent parental management strategies. In contrast, common barriers involved child resistance, poor sensory acceptance, and caregiver inconsistency. These findings highlight the importance of improving product acceptability and providing practical, family-centered support through educational tools, clear guidance, and tailored transition plans. Involving role models, ensuring sufficient clinical time, and actively engaging schools could further help address challenges and support smooth transitions.

This study provides a comprehensive overview of global practices of health professionals’ protein substitute transition practices in PKU. Its broad international scope enhances the relevance of the findings. However, several limitations should be acknowledged. The questionnaire was not validated, and European respondents were overrepresented (67%), with limited participation from Asia, North America, South America, and Oceania, and no responses from Africa. Some countries were represented by only one participant, and the distribution within continents was uneven, with the UK accounting for almost a quarter of European responses. This imbalance may limit the generalizability of the findings to underrepresented regions or countries with different healthcare systems, reimbursement structures, and product availability. As with any survey-based research, responses may have been influenced by recall bias or differences in interpretation. Variability in terminology and English proficiency could have impacted how some questions were understood. Additionally, while all respondents were dietitians or physicians, specific professional roles were not recorded, limiting role-based analysis. The study did not collect patient-level clinical outcome data, which was beyond its scope, and did not explore detailed monitoring strategies (e.g., clinical or biochemical), which could be considered in future research. Breastfeeding practices were also outside the scope of this survey and are highlighted as a topic for future investigation. Despite these limitations, the study provides valuable insight into global practice patterns and lays a foundation for future improvement. There is a clear need for consistent, accessible, and adaptable strategies to better support patients, families and professionals worldwide during the transition process.

## 5. Conclusions

This global survey shows wide variation in the timing, formats, and strategies used to transition protein substitutes in children with PKU. These differences are mainly shaped by product availability, local protocols, and health system factors. Although flexibility is important to meet individual and regional needs, the findings highlight the importance of clear and practical guidance to support families and healthcare professionals throughout the transition process. Expanding access to a broader range of protein substitutes across all regions is important to ensure more equitable care. Active involvement of schools can also help ease transitions by providing structure, supervision, and support. The development of practical, family-centered resources and structured educational tools can improve confidence and consistency during these critical stages. These findings support clear clinical recommendations, including initiating transition in line with developmental milestones, improving access to varied and age-appropriate protein substitute formulations, actively engaging educational settings, and providing structured, family-centred resources to support adherence. Further research should explore the long-term outcomes of differences in transition practices and identify effective ways to support families and professionals in a variety of clinical settings.

## Figures and Tables

**Table 1:** Transition practices from first-stage Phe-free infant formula to second-stage protein substitutes by continent.

	Continents					
	Europe (*n*, %)	Asia (*n*, %)	North America (*n*, %)	South America (*n*, %)	Oceania (*n*, %)	Total (*n*, %)
Discontinuation Age of First-Stage Phe-free Infant Formula						
3–5 months old	1 (1%)	0 (0%)	0 (0%)	0 (0%)	0 (0%)	1 (1%)
6–12 months old	14 (20%)	6 (50%)	0 (0%)	0 (0%)	3 (100%)	23 (22%)
1–2 years old	47 (67%)	5 (42%)	9 (82%)	7 (100%)	0 (0%)	68 (66%)
>2 years old	8 (12%)	1 (8%)	2 (18%)	0 (0%)	0 (0%)	11 (11%)
Total (*n*)	70	12	11	7	3	103
Introduction Age of Second-Stage PS						
3–5 months old	10 (14%)	0 (0%)	0 (0%)	0 (0%)	0 (0%)	10 (10%)
6–12 months old	43 (61%)	4 (31%)	3 (27%)	0 (0%)	3 (100%)	53 (50%)
1–2 years old	10 (14%)	4 (31%)	4 (36%)	8 (100%)	0 (0%)	26 (25%)
>2 years old	7 (10%)	5 (38%)	4 (36%)	0 (0%)	0 (0%)	16 (15%)
Total (*n*)	70	13	11	8	3	105
Factors Influencing Timing of Second-Stage PS Introduction ^1^						
Weaning alignment	39 (60%)	1 (8%)	4 (36%)	0 (0%)	2 (67%)	46 (46%)
Child nutritional requirements	26 (40%)	4 (33%)	7 (64%)	3 (38%)	2 (67%)	42 (42%)
Better acceptance with age	10 (15%)	2 (17%)	3 (27%)	0 (0%)	0 (0%)	15 (15%)
Clinical protocols and instructions	4 (6%)	1 (8%)	0 (0%)	4 (50%)	0 (0%)	9 (9%)
Reimbursement policies	2 (3%)	2 (17%)	0 (0%)	1 (12%)	0 (0%)	5 (5%)
Total (*n*)	65	12	11	8	3	99
Most Common Type of Second-Stage PS						
Powder (drink)	29 (41%)	8 (62%)	9 (82%)	8 (100%)	1 (33%)	55 (52%)
Powder (semi-solid)	40 (56%)	3 (23%)	1 (9%)	0 (0%)	2 (67%)	46 (43%)
Ready-to-drink liquid	2 (3%)	1 (8%)	0 (0%)	0 (0%)	0 (0%)	3 (3%)
Ready-to-use semi-solid	0 (0%)	1 (8%)	1 (9%)	0 (0%)	0 (0%)	2 (2%)
Total (*n*)	71	13	11	8	3	106

^1^ Open-ended responses were thematically coded; multiple themes per response were possible, so percentages may exceed 100%. Phe: phenylalanine; PS: protein substitute. Note: Some participants did not respond to all survey questions, so the number of responses varies across items, and totals may not always add up to 100% due to rounding.

**Table 2:** Transition practices from second-stage to third-stage protein substitutes by continent.

	Continents					
	Europe (*n*, %)	Asia (*n*, %)	North America (*n*, %)	South America (*n*, %)	Oceania (*n*, %)	Total (*n*, %)
Introduction Age of Third-Stage PS						
1–2 years old	3 (4%)	1 (8%)	2 (18%)	0 (0%)	1 (33%)	7 (7%)
3–5 years old	36 (53%)	6 (50%)	2 (18%)	1 (12%)	1 (33%)	46 (45%)
6–10 years old	20 (30%)	2 (17%)	4 (36%)	5 (63%)	1 (33%)	32 (31%)
>10 years old	7 (10%)	2 (17%)	3 (27%)	2 (25%)	0 (0%)	14 (14%)
Depends	2 (3%)	1 (8%)	0 (0%)	0 (0%)	0 (0%)	3 (3%)
Total (*n*)	68	12	11	8	3	102
Factors Influencing Timing of Third-Stage PS Introduction ^1^						
Nutritional and clinical considerations	26 (38%)	3 (25%)	10 (91%)	4 (50%)	0 (0%)	43 (42%)
Practical and child behavioral reasons	32 (47%)	3 (25%)	3 (27%)	2 (25%)	1 (33%)	41 (40%)
Protocols and manufacturer instructions	13 (19%)	3 (25%)	1 (9%)	3 (38%)	0 (0%)	20 (20%)
Taste and flavor preferences	9 (13%)	1 (8%)	3 (27%)	1 (13%)	0 (0%)	14 (14%)
Availability of products	1 (1%)	3 (25%)	0 (0%)	0 (0%)	1 (33%)	5 (5%)
Reimbursement policies	2 (3%)	1 (8%)	0 (0%)	1 (13%)	1 (33%)	5 (5%)
Total (*n*)	68	12	11	8	3	102
Most Common Types of Third-Stage PS ^2^						
Ready-to-drink liquid	60 (85%)	11 (92%)	8 (73%)	0 (0%)	3 (100%)	82 (78%)
Powder (drink)	32 (45%)	6 (50%)	10 (91%)	8 (100%)	1 (33%)	57 (54%)
Tablets	8 (11%)	0 (0%)	0 (0%)	1 (13%)	0 (0%)	9 (9%)
Bars	4 (6%)	1 (8%)	0 (0%)	0 (0%)	0 (0%)	5 (5%)
Powder (semi-solid)	4 (6%)	1 (8%)	0 (0%)	0 (0%)	0 (0%)	5 (5%)
Granules/microgranules	2 (3%)	0 (0%)	0 (0%)	0 (0%)	0 (0%)	2 (2%)
Total (*n*)	71	12	11	8	3	105

^1^ Open-ended responses were thematically coded; multiple themes per response were possible, so percentages may exceed 100%. ^2^ Multiple response question. PS: protein substitute. Note: Some participants did not respond to all survey questions, so the number of responses varies across items, and totals may not always add up to 100% due to rounding.

**Table 3:** Duration of transition from second-stage to third-stage protein substitutes by continent.

	Continents					
	Europe (*n*, %)	Asia (*n*, %)	North America (*n*, %)	South America (*n*, %)	Oceania (*n*, %)	Total (*n*, %)
Duration of Transition						
<1 month	23 (38%)	3 (27%)	4 (36%)	1 (12%)	0 (0%)	31 (33%)
1–2 month	11 (18%)	4 (37%)	4 (36%)	3 (38%)	2 (67%)	24 (26%)
3–6 month	14 (23%)	3 (27%)	1 (9%)	3 (38%)	0 (0%)	21 (23%)
7–12 month	2 (3%)	0 (0%)	1 (9%)	0 (0%)	1 (33%)	4 (4%)
1–3 years	4 (7%)	0 (0%)	0 (0%)	1 (12%)	0 (0%)	5 (5%)
Depends	7 (11%)	1 (9%)	1 (9%)	0 (0%)	0 (0%)	9 (10%)
Total (*n*)	61	11	11	8	3	94

Note: Some participants did not respond to all survey questions, so the number of responses varies across items, and totals may not always add up to 100% due to rounding.

**Table 4:** Factors influencing the choice of third-stage protein substitutes by continent.

	Continents					
	Europe (*n*, %)	Asia (*n*, %)	North America (*n*, %)	South America (*n*, %)	Oceania (*n*, %)	Total (*n*, %)
Influencing Factors ^1^						
Patient preference	47 (66%)	5 (38%)	10 (91%)	4 (50%)	3 (100%)	69 (65%)
Convenience of minimal preparation	50 (70%)	8 (62%)	5 (45%)	3 (38%)	3 (100%)	69 (65%)
Nutritional composition of products	35 (49%)	7 (54%)	9 (82%)	3 (38%)	1 (33%)	55 (52%)
Reimbursement policies	17 (24%)	5 (38%)	4 (36%)	4 (50%)	2 (67%)	32 (30%)
Child’s age and maturity	26 (37%)	4 (31%)	0 (0%)	1 (13%)	0 (0%)	31 (29%)
Availability or access to products	13 (18%)	4 (31%)	1 (9%)	6 (75%)	1 (33%)	25 (24%)
Parental choice	13 (18%)	3 (23%)	2 (18%)	0 (0%)	0 (0%)	18 (17%)
Products listed on the hospital formulary	2 (3%)	2 (15%)	1 (9%)	3 (38%)	0 (0%)	8 (8%)
Other factors ^2^	1 (1%)	0 (0%)	1 (9%)	2 (25%)	0 (0%)	4 (4%)
Total (*n*)	71	13	11	8	3	106

^1^ Top-three answers question. ^2^ Other factors included experience with older sibling, packaging, and product price. Note: Percentages may not total 100% due to rounding.

**Table 5:** Subsequent changes in third-stage protein substitutes.

	*n*	%
Subsequent change to a different third-stage PS (*n* = 106)		
Yes	55	52
No	51	48
Reasons for change (*n* = 55) ^1^		
Child resistance due to taste, smell, and texture	44	80
Child resistance due to high volume	31	56
Supply issues	27	49
Gastrointestinal problems	24	44
Specific age indication	18	33
Deterioration in metabolic control	16	29
Other	8	15

^1^ Multiple-response question. PS: protein substitute.

**Table 6:** Prescription of cGMP-based protein substitutes and factors considered during transition to third-stage protein substitutes.

	*n*	%
cGMP prescription when transitioning from second- to third-stage PS (*n* = 106)		
Yes	65	61
No	14	13
Not available in country	27	26
Factors considered for cGMP prescription (*n* = 74) ^1,2^		
Patient preference	32	43
Phe tolerance of patients	24	32
Compliance issues with AA-based PS	23	31
Nutritional and physiological advantages of cGMP ^3^	18	24
Better taste, smell, and/or palatability of cGMP	14	19
Metabolic control of patients	10	14
Practicality	4	5
Gastrointestinal symptoms	4	5
Patient age	3	4
Better acceptability	3	4

^1^ Multiple-response question. ^2^ *n* = 27/106 (26%) of professionals who reported that cGMP protein substitutes are not available in their country, and *n* = 5 (5%) who did not provide additional details, were excluded from the analysis. ^3^ Nutritional and physiological advantages refer to factors such as effects on microbiota, improved bioavailability, extra calorie provision, and being a source of natural protein. AA: amino acid; cGMP: casein-glycomacropeptide; Phe: phenylalanine; PS: protein substitute.

**Table 7:** Facilitators and barriers in the transition from second- to third-stage protein substitutes.

Facilitators and Barriers to Introducing Third-Stage PS (*n* = 106) ^1^	*n*	%
Facilitators		
Motivation of child/parents	84	79
Better taste, smell, or texture of the third-stage PS	73	69
Parental management strategies ^2^	60	57
Child age and maturity	52	49
Ease of preparation	50	47
Increased independence	32	30
Seeing other children/older people take PS at PKU events	29	27
Parents’ educational level	24	23
Role models (sibling/cousin/friend) with PKU	23	22
Parents prior knowledge about the transition process	22	21
Written individual plan for parents	15	14
Poor experience with the second-stage PS	10	9
Written plan for nursery	6	6
Barriers		
Aversion to a new PS	74	70
Unappealing taste, smell, or texture	73	69
Lack of parental consistency/persistence	34	32
Parental fear of change	33	31
Poor child behavior	26	25
High volume of the PS	25	24
Lack of engagement by parents/carers	14	13
Poor understanding of the process by parents/carers	12	11
Lack of supportive materials	8	8
Inadequate dietetic time to support families	8	8

^1^ Top-three answers question. ^2^ e.g., consistency, establishing a time routine, persistent with a resolute approach, small rewards, calm/positive approach. PKU: phenylketonuria; PS: protein substitute.

## Data Availability

The original contributions presented in this study are included in the article. Further inquiries can be directed to the corresponding author.

## Supplementary Materials

The following supporting information can be downloaded at: https://www.mdpi.com/article/10.3390/nu17162650/s1, Supplementary File S1: Survey questionnaire.

## Abbreviations

The following abbreviations are used in this manuscript:

PKU	Phenylketonuria
PAH	Phenylalanine hydroxylase
Phe	Phenylalanine
cGMP	Casein-glycomacropeptide

## References

[1] van Spronsen F.J., Blau N., Harding C., Burlina A., Longo N., Bosch A.M. (2021). Phenylketonuria. Nat. Rev. Dis. Primers.

[2] Scriver C.R. (2007). The PAH gene, phenylketonuria, and a paradigm shift. Hum. Mutat..

[3] Mitchell J.J., Trakadis Y.J., Scriver C.R. (2011). Phenylalanine hydroxylase deficiency. Genet. Med..

[4] Thomas L., Olson A., Romani C. (2023). The impact of metabolic control on cognition, neurophysiology, and well-being in PKU: A systematic review and meta-analysis of the within-participant literature. Mol. Genet. Metab..

[5] Vockley J., Andersson H.C., Antshel K.M., Braverman N.E., Burton B.K., Frazier D.M., Mitchell J., Smith W.E., Thompson B.H., Berry S.A. (2014). Phenylalanine hydroxylase deficiency: Diagnosis and management guideline. Genet. Med..

[6] Thomas L.S. (2022). Neuropsychological Assessment in Inherited Metabolic Diseases: Tracking Outcomes in Phenyketonuria (PKU). Ph.D. Thesis.

[7] Hillert A., Anikster Y., Belanger-Quintana A., Burlina A., Burton B.K., Carducci C., Chiesa A.E., Christodoulou J., Đorđević M., Desviat L.R. (2020). The genetic landscape and epidemiology of phenylketonuria. Am. J. Hum. Genet..

[8] Burgard P., Lachmann R.H., Walter J.H. (2022). Hyperphenylalaninaemia. Inborn Metabolic Diseases: Diagnosis and Treatment.

[9] Manta-Vogli P.D., Dotsikas Y., Loukas Y.L., Schulpis K.H. (2020). The phenylketonuria patient: A recent dietetic therapeutic approach. Nutr. Neurosci..

[10] McWhorter N., Ndugga-Kabuye M.K., Puurunen M., Ernst S.L. (2022). Complications of the low phenylalanine diet for patients with phenylketonuria and the benefits of increased natural protein. Nutrients.

[11] Singh R.H., Rohr F., Frazier D., Cunningham A., Mofidi S., Ogata B., Splett P.L., Moseley K., Huntington K., Acosta P.B. (2014). Recommendations for the nutrition management of phenylalanine hydroxylase deficiency. Genet. Med..

[12] González-Lamuño D., Morencos C., Arrieta F.J., Venegas E., Vicente-Rodríguez G., Casajús J.A., Couce M.L., Aldámiz-Echevarría L. (2024). Supplementation for performance and health in patients with phenylketonuria: An exercise-based approach to improving dietary adherence. Nutrients.

[13] Firman S., Witard O.C., O’Keeffe M., Ramachandran R. (2021). Dietary protein and protein substitute requirements in adults with phenylketonuria: A review of the clinical guidelines. Clin. Nutr..

[14] Belanger-Quintana A., Dokoupil K., Gokmen-Ozel H., Lammardo A., MacDonald A., Motzfeldt K., Nowacka M., Robert M., Van Rijn M., Ahring K. (2012). Diet in phenylketonuria: A snapshot of special dietary costs and reimbursement systems in 10 international centers. Mol. Genet. Metab..

[15] Guest J., Bai J.-J., Taylor R., Sladkevicius E., Lee P., Lachmann R. (2013). Costs and outcomes over 36 years of patients with phenylketonuria who do and do not remain on a phenylalanine-restricted diet. J. Intellect. Disabil. Res..

[16] Pena M., De Almeida M., Van Dam E., Ahring K., Bélanger-Quintana A., Dokoupil K., Gokmen-Ozel H., Lammardo A., MacDonald A., Robert M. (2016). Protein substitutes for phenylketonuria in Europe: Access and nutritional composition. Eur. J. Clin. Nutr..

[17] Keskin F.N., Şahin T.Ö., Capasso R., Ağagündüz D. (2022). Protein substitutions as new-generation pharmanutrition approach to managing phenylketonuria. Clin. Exp. Pediatr..

[18] Wang B., Xia Y., Cheng M., Luo H., Xue L., Gong A., Liu X., Liao G., Song J., Ning K. (2024). Insurance reimbursement for special foods and phenylalanine levels in patients with PKU in China. JAMA Netw. Open.

[19] Hagedorn T.S., van Berkel P., Hammerschmidt G., Lhotáková M., Saludes R.P. (2013). Requirements for a minimum standard of care for phenylketonuria: The patients’ perspective. Orphanet J. Rare Dis..

[20] Eijgelshoven I., Demirdas S., Smith T.A., van Loon J.M., Latour S., Bosch A.M. (2013). The time consuming nature of phenylketonuria: A cross-sectional study investigating time burden and costs of phenylketonuria in the Netherlands. Mol. Genet. Metab..

[21] Martins A.M., Pessoa A.L.S., Quesada A.A., Ribeiro E.M. (2020). Unmet needs in PKU and the disease impact on the day-to-day lives in Brazil: Results from a survey with 228 patients and their caregivers. Mol. Genet. Metab. Rep..

[22] Wang L., Zou H., Ye F., Wang K., Li X., Chen Z., Chen J., Han B., Yu W., He C. (2017). Household financial burden of phenylketonuria and its impact on treatment in China: A cross-sectional study. J. Inherit. Metab. Dis..

[23] Vargas P.R., Poubel M., Martins B., Velez P., Vilela D., Mesojedovas D., Magalhães T.S.P.C., Louie K.S., Pessoa A. (2025). Patient journey and disease burden characterization of the population with phenylketonuria (PKU) in Brazil: A retrospective analysis through data reported in the public health system administrative database (DATASUS). Lancet Reg. Health-Am..

[24] Sabaté E. (2003). Adherence to Long-Term Therapies: Evidence for Action.

[25] Evans S., Daly A., Wildgoose J., Cochrane B., Chahal S., Ashmore C., Loveridge N., MacDonald A. (2019). How does feeding development and progression onto solid foods in PKU compare with non-PKU children during weaning?. Nutrients.

[26] Yilmaz O., Pinto A., Daly A., Ashmore C., Evans S., Yabanci Ayhan N., MacDonald A. (2022). Transitioning of protein substitutes in patients with phenylketonuria: Evaluation of current practice. Orphanet J. Rare Dis..

[27] Yilmaz Nas O., Ashmore C., Evans S., Pinto A., Daly A., Yabanci Ayhan N., MacDonald A. (2025). Transitioning of protein substitutes in patients with phenylketonuria: A pilot study. Front. Nutr..

[28] DATAtabTeam (2024). DATAtab: Online Statistics Calculator.

[29] Pinto A., Adams S., Ahring K., Allen H., Almeida M., Garcia-Arenas D., Arslan N., Assoun M., Altınok Y.A., Barrio-Carreras D. (2019). Weaning practices in phenylketonuria vary between health professionals in Europe. Mol. Genet. Metab. Rep..

[30] Evans S., Daly A., Wildgoose J., Cochrane B., Ashmore C., Kearney S., MacDonald A. (2019). Mealtime anxiety and coping behaviour in parents and children during weaning in PKU: A case-control study. Nutrients.

[31] Giovannini M., Riva E., Salvatici E., Cefalo G., Radaelli G. (2014). Randomized controlled trial of a protein substitute with prolonged release on the protein status of children with phenylketonuria. J. Am. Coll. Nutr..

[32] Giarratana N., Gallina G., Panzeri V., Frangi A., Canobbio A., Reiner G. (2018). A new Phe-free protein substitute engineered to allow a physiological absorption of free amino acids for phenylketonuria. J. Inborn Errors Metab. Screen..

[33] Russo G.L., Puleo S., Cavella S., Scala I., Fidaleo M., Di Monaco R. (2025). Advancements in food science for Phenylketonuria (PKU) management: A comprehensive review. Crit. Rev. Food Sci. Nutr..

[34] Jones H., Pinto A., Evans S., Ford S., O’Driscoll M., Buckley S., Ashmore C., Daly A., MacDonald A. (2021). Provision and supervision of food and protein substitute in school for children with PKU: Parent experiences. Nutrients.

[35] Zwiesele S., Bannick A., Trepanier A. (2015). Parental strategies to help children with phenylketonuria (PKU) cope with feeling different. Am. J. Med. Genet. Part A.

