# Supplementary material for: Global Clinical Practice in Transitioning Protein Substitutes for Children with Phenylketonuria
Source: Nutrients. 2025 Aug 15;17(16):2650. doi: 10.3390/nu17162650 (PMC12389518; doi:10.3390/nu17162650)
Supplement: Supplementary file 1 [file nutrients-17-02650-s001.zip › nutrients-3794115-supplementary.pdf]

Supplementary File S1: Survey questionnaire.

Transition from Second Stage to Third Stage Protein Substitute in PKU

Dear Colleagues,

Thank you for your help in completing this survey about your experiences in transitioning patients with PKU from the second stage to third stage protein substitutes in childhood. With this information, we hope to understand the different practices and give guidance about how to support patients, their parents/caregivers and healthcare professionals involved in this process. The survey will take approximately 10-15 minutes to complete.

If you require any further information, please do not hesitate to contact us.

Best wishes,

Ozlem Yilmaz

o.yilmaz@ybu.edu.tr

Research Dietitian in IMD

Ankara Yildirim Beyazit University

Prof. Anita MacDonald

Anita.Macdonald@nhs.net

Consultant Dietitian in IMD

Birmingham Children’s Hospital

* Indicates required question

Types of Protein Substitutes

The following information helps describe the different types of protein substitutes referred to in the questionnaire :

First stage

- Infant phenylalanine-free formula (e.g. PKU Anamix infant, PKU Start)

Second stage

- Suitable from the age of 6 months or 1 year
- Weaning amino acid protein substitutes, given as a semi-solid consistency from a spoon (e.g. PKU Anamix First spoon, PKU gel, PKU explore)
- Powder amino acid supplements given as a drink (e.g. PKU Anamix Junior)
- Liquid amino acid supplements (e.g. PKU Anamix Junior LQ)

Third stage

- Suitable from the age of 3 or 4 years or older
- Liquid amino acid protein substitutes (e.g. PKU Air, PKU Cooler, PKU Lophlex LQ)
- Powder amino acid protein substitutes (e.g. PKU Express, PKU Lophlex powder)
- Casein glycomacropeptide (cGMP) protein substitutes (e.g. Glytactin Bettermilk, GMPro, PKU Sphere)
- Slow-release protein substitute (granules/microtablets – e.g. PKU Golike, PKU Microtabs)

1. **Which one of the following statements applies to your center:** *

   Mark only one oval.

   - ( ) We manage adults and children *Skip to question 2*
   - ( ) We only manage children *Skip to question 2*
   - ( ) We only manage adults (≥18 years of age)

2. **Please provide your center's location, including city and country.** (Please do not use abbreviations.)

   ____

3. **What is the total number of all your patients with PKU in your center?** *

   ____

4. **What is the approximate number of patients with PKU aged 16 years or under in your center?**

   ____

5. **What is the most common age at which a phenylalanine-free infant formula (first stage protein substitute) is stopped/discontinued for children with PKU in your care?**

   Mark only one oval.

   - ( ) 3-5 months old
   - ( ) 6-12 months old
   - ( ) 1-2 years
   - ( ) >2 years
   - ( ) Other: ________________________________

6. **What is the most common age at which a second stage protein substitute is introduced for children with PKU in your care?**

Mark only one oval.

- 3-5 months old
- 6-12 months old
- >1-2 years
- >2 years
- Other: ____________________

7. **Why is this age chosen?** *

____
____
____
____
____

8. **What is the most common type of second stage protein substitute used when it is time to move on from a phenylalanine-free infant formula (first stage protein substitute)?**

Mark only one oval.

- Powder made into a semi-solid
- Powder made into a drink
- Ready to drink liquid
- Ready to use semi-solid

9. **What is the most common age at which a third stage protein substitute is introduced fo[illegible] children with PKU in your care?**

Mark only one oval.

- 1-2 years old
- 3-5 years old
- 6-10 years old
- >10 years old
- Other: ____________________

10. **Why is this age chosen?** *

____
____
____
____
____

11. **Which healthcare professional is primarily responsible for leading the transition process from second stage to third stage protein substitute?**

Mark only one oval.

- ( ) Dietitian
- ( ) Nurse
- ( ) Doctor
- ( ) Other: _______________________

12. **After transitioning to a third stage protein substitute in PKU, would your patients usually change again at a later age to a different type of protein substitute?** [Please answer 'No' if the only product changes are changes in pack size (e.g. cooler 10 to cooler 15/lophlex10 to lophlex20) or changes in a flavour]

Mark only one oval.

- ( ) Yes *Skip to question 13*
- ( ) No *Skip to question 14*

13. **Please choose all the factors that would lead to a change to a different type of third stage protein substitute.** Please tick all the answers that apply.

Tick all that apply.

- [ ] Child resistance due to taste, smell, and texture of protein substitute
- [ ] Child resistance due to high volume of protein substitute
- [ ] Deterioration in blood phenylalanine control
- [ ] Gastrointestinal problems with new protein substitute
- [ ] Supply issues with new protein substitute
- [ ] Specific age indication of protein substitute
- [ ] Other: _______________________

14. **What type of third stage protein substitute is the one most often used by your PKU patient group?**

Tick all that apply.

- [ ] Ready to drink liquid
- [ ] Powder made into a drink
- [ ] Powder made into a semi solid consistency (like a dessert)
- [ ] Bar
- [ ] Tablets
- [ ] Other: ______________________

15. **Please choose the three most important factors influencing choice of third stage protein substitute?**

Tick all that apply.

- [ ] Parents
- [ ] Experience with older sibling
- [ ] Ready to use /minimal preparation
- [ ] Packaging
- [ ] Child age/maturity
- [ ] Patient choice
- [ ] Nutritional composition of products
- [ ] Availability of samples
- [ ] Products that are reimbursed in your country/state
- [ ] Product price
- [ ] Products available on the hospital formulary
- [ ] Other: ______________________

16. **Do you prescribe GMP protein substitutes to patients with PKU when transitioning fro[illegible] a second stage to a third stage protein substitute?**

Mark only one oval.

- ( ) Yes
- ( ) No
- ( ) N/A - GMP products not available *Skip to question 18*

17. **If GMP products are available in your country, please briefly explain the factors you consider when deciding if to prescribe GMP protein substitutes during the transition to third-stage protein substitute.**

Please describe the transition process from a second stage to a third stage protein substitute in your PKU patients.

18. **Average child age of transition** *

19. **Average duration of transition** *

20. **Approach (step by step or immediate changeover). Please provide any relevant details**

21. **Follow up (phone/video call, clinics, home visits,…)** *

22. **Are you usually able to engage the help of nursery/school staff when introducing a third stage protein substitute?**

Mark only one oval.

- Yes
- No

23. **Please provide further details**

____

24. **Please rate from one to five how you find the transition from a second stage to a third stage protein substitute for most of your patients with PKU?**

Mark only one oval.

	1	2	3	4	5	
very	◯	◯	◯	◯	◯	very difficult

25. **Choose the 3 most common barriers you face when introducing a third stage protein substitute?**

Tick all that apply.

- [ ] Lack of engagement by parents/carers
- [ ] Lack of consistency /persistence of parents/carers
- [ ] Poor understanding of the process by parents/carers
- [ ] Parental fear of change
- [ ] Lack of supportive materials
- [ ] Taste, smell, or texture of the third stage protein substitute
- [ ] High volume of the third stage protein substitute
- [ ] Aversion to a new type of protein substitute
- [ ] Poor child behaviour
- [ ] Inadequate dietetic time to support families
- [ ] Other: ______________________________

26. **What do you consider to be important facilitators in helping the transition process?** Please select all that apply.

Tick all that apply.

- [ ] Child age and maturity
- [ ] Motivation of child/parents/carers
- [ ] Parents educational level
- [ ] Parents prior knowledge about the transition process
- [ ] Parental management strategies (e.g., consistency, establishing a time routine, persistent with a resolute approach, small rewards, calm/positive approach)
- [ ] A role model (sibling/cousin/friend) with PKU
- [ ] Seeing other children/older people take protein substitutes at PKU events
- [ ] Poor experience with the second stage protein substitute
- [ ] Ease of preparation of the third stage protein substitute
- [ ] Increased independence with the third stage protein substitute
- [ ] Better taste, smell, or texture of the third stage protein substitute
- [ ] Having a written individual health care plan for parents/caregivers
- [ ] Having a written individual health care plan for nursery
- [ ] Other: ______________________

27. **Is the transition of the protein substitute process the same for all disorders of protein metabolism?**

Mark only one oval.

- ( ) Yes
- ( ) No

28. **If your answer is 'No', please explain what the differences are between the different disorders**

____

____

____

____

____

29. **What do you feel is currently missing to support children and their parents/carers to aid the transition from a second stage to a third stage protein substitute?**

Tick all that apply.

- [ ] Guidance books
- [ ] Child rewards/sticker charts
- [ ] Educational videos
- [ ] Step by step chart or books
- [ ] Other: ______________________

30. **Is there anything else that would help you support your patients/care givers with the transition from a second stage to a third stage protein substitute?**

____

____

____

____

____

This content is neither created nor endorsed by Google.

Google Forms
